# A User Authentication Scheme Based on Elliptic Curves Cryptography for Wireless Ad Hoc Networks

**DOI:** 10.3390/s150717057

**Published:** 2015-07-14

**Authors:** Huifang Chen, Linlin Ge, Lei Xie

**Affiliations:** 1 Department of Information Science and Electronic Engineering, Zhejiang University, Hangzhou 310027, China; E-Mails: lynkeh13@zju.edu.cn (L.G.); xiel@zju.edu.cn (L.X.); 2 Zhejiang Provincial Key Laboratory of Information Network Technology, Hangzhou 310027, China

**Keywords:** wireless ad hoc network (WANET), self-certified public key (SCPK), elliptic curves cryptography (ECC), user authentication, session key agreement

## Abstract

The feature of non-infrastructure support in a wireless ad hoc network (WANET) makes it suffer from various attacks. Moreover, user authentication is the first safety barrier in a network. A mutual trust is achieved by a protocol which enables communicating parties to authenticate each other at the same time and to exchange session keys. For the resource-constrained WANET, an efficient and lightweight user authentication scheme is necessary. In this paper, we propose a user authentication scheme based on the self-certified public key system and elliptic curves cryptography for a WANET. Using the proposed scheme, an efficient two-way user authentication and secure session key agreement can be achieved. Security analysis shows that our proposed scheme is resilient to common known attacks. In addition, the performance analysis shows that our proposed scheme performs similar or better compared with some existing user authentication schemes.

## 1. Introduction

Wireless ad hoc network (WANET) is a decentralized type of wireless network. It has widely practical applications, such as tactical communication, emergency communication, temporary communication, and so on. However, the WANET is vulnerable to various attacks due to the absence of infrastructure support [1]. Security of the WANET is critical for its deployment and management. Moreover, the user authentication is the first safety barrier in a network. That is, each node needs to ensure that the peer node with which it is communicating is he/she claims. On the other hand, wireless devices have limited computation capability, memory and energy. For the resource-constrained WANET, an efficient and lightweight user authentication scheme is necessary.

Many user authentication schemes have been proposed for the WANET in recent years. In [2], Bechler, M. *et al*. proposed a cluster-based user authentication scheme, where a cluster head controls the cluster. Since the cluster structure is useful for enhancing the scalability, the cluster-based authentication scheme is more suitable for large-scale networks. However, this scheme is exposed to the single point of failure since all cluster members depend on the cluster head. A distributed key management and user authentication approach is proposed in [3], where the concepts of identity-based key cryptography and threshold secret sharing are used. This approach works in a self-organizing way to provide the key generation and management service, and effectively solves the single point of failure problem. However, the security is breached when a threshold number of shareholders are compromised. Other user authentication schemes were proposed in [4] and [5], where a certificate server (CS) is used to issue user’s certificate and public key. In addition, users perform the identity authentication with the assistance of CS. However, the CS is hard to be set up because of the dynamics of nodes in WANETs. Moreover, if the identity authentication needs the help of CS, the storage and management requirements of certificates increase the burden for CS.

Most user authentication schemes mentioned above use the public key infrastructure (PKI) [6] or the identity-based public key cryptosystem (ID-PKC) [7]. However, the high complexity for certificates in PKI increases the system burden greatly. In addition, the key escrow problem of ID-PKC is also a serious problem.

Unlike the prior work, the self-certified public key (SCPK) cryptosystem [8] is another kind of scheme. In this scheme, certificate authority (CA) embeds its signature in user’s public key, and computes user’s private key cooperatively with users. The advantage of the SCPK scheme is that the authenticity of a user’s public key can be verified publicly without using any certificate issued by the CA and the private key known to the user only. Hence, this scheme does not need the digital certificates as in the PKI scheme, as well as avoids the key escrow problem of the ID-PKC scheme.

Compared with RSA, one of most widely accepted and traditional public key cryptographies, elliptic curves cryptography (ECC), has attracted considerable attention due to its smaller key size and lower resource consumption for achieving the same security level. This is because the addition operation in ECC is the counterpart of modular multiplication in RSA, and multiple addition is the counterpart of modular exponentiation. Furthermore, ECC is based on the intractability of the elliptic curve discrete logarithm problem (ECDLP). That is, finding an effective and rapid solution to the ECDLP is still a hard problem [9].

Hence, the user authentication scheme based on SCPK and ECC is a feasible alternative for resource-constrained wireless networks, such as WANET, mobile ad hoc networks and wireless sensor networks. Several user authentication schemes using SCPK and ECC have been proposed [10,11,12]. In [10], a distributed user authentication scheme based on SCPK was presented. In this scheme, each user gets his/her public/private key from CA through a secure communication channel. However, providing a secure communication channel in a wireless network is not a trivial thing. A user authentication and key agreement scheme was proposed in [11], where the timestamp mechanism is used to resist the replay attack. However, it is a difficult task to maintain time synchronization in a WANET. In addition, the session key cannot resist key compromise impersonation attack in this scheme. In [12], a novel self-certified secure access authentication protocol was proposed. In this scheme, a challenge-response mechanism is adopted to resist the replay attack. However, the user’s private key can be compromised easily.

In this paper, we propose a user authentication scheme based on SCPK and ECC for a WANET. In order to reduce the computational complexity, the SCPK proposed in [13] is modified using ECC. The proposed user authentication scheme consists of three phases, namely the setup phase, the user registration phase, and the user authentication phase. CA selects and generates the global system parameters, and publishes them to the whole network in the setup phase. Users register with CA to obtain the private/public key pairs for authentication in the user registration phase. In the user authentication phase, users complete their identities authentication using their private/public keys and the CA’s public key. Finally, we analyze the performance of the proposed user authentication scheme, in terms of the security, the storage overhead, the communication overhead and the computation overhead. Analysis results show that our proposed scheme achieves efficient two-way user authentication and secure session key agreement. Hence, the proposed scheme is efficient, and suitable for the resource-constrained WANET.

Our proposed user authentication scheme differs from other existing user authentication schemes in [10,11,12] are: (1) A secure communication channel for distributing user’s public/private key does not need; (2) A modified challenge-response mechanism is adopted to resist the replay attack; (3) The authentication mechanism between user and CA in the user registration phase is used to resist the user masquerade attack.

The remainder paper is organized as follows. In Section 2, the system model for the proposed user authentication scheme is introduced. In Section 3, the proposed user authentication scheme based on SCPK and ECC is presented. The security and performance of the proposed scheme are analyzed in Section 4 and Section 5, respectively. Finally, we conclude the paper in Section 6.

## 2. System Model

**Figure 1:**
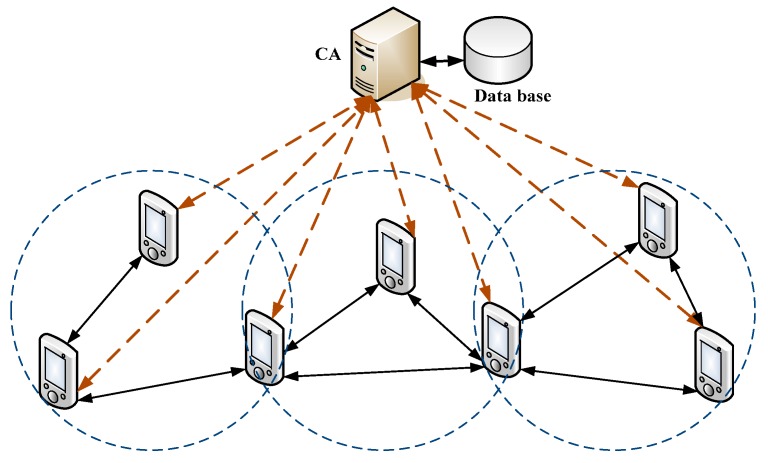
The system architecture of the proposed user authentication scheme in a wireless ad hoc network (WANET).

Figure 1 shows the system architecture for our proposed user authentication scheme.

In this system, a CA is deployed to generate user’s private/public key pairs cooperatively with users. Each user knows the public key of the CA. With the public key of CA, each user can verify the peer user’s identity with whom he/she is communicating.

To clarify the proposed user authentication scheme, notations and their denotations are summarized in Table 1.

**Table 1:** Notations and their denotations.

Notations	Denotations
*p*	A large prime number
GF(*p*)	The finite field
*a*, *b*	The elliptic curve parameters, real numbers
*E_p_*(*a*, *b*)	The elliptic curve over GF(*p*) consisting of the elliptic group of points defined by $y^{2}=x^{3}+ax+b\;(\mathrm{mod}p)$, where $(4a^{3}+27b^{2})\mathrm{mod}p\neq 0$
*G*	A base point (*x*, *y*) selected on *E_p_*(*a*, *b*) with a large order
*n*	The order of point *G*, where *n* is the smallest positive integer such that nG=O (infinity point), and *n* is a large prime number
*SHA*(•)	A one-way hash function
*s*_CA_	The private key of CA
*P*_CA_	The public key of CA
*N*_CA_	A nonce randomly generated by CA from [2, *n*−2]
*N*_i_	A nonce randomly generated by U_i_ from [2, *n*−2]
*s*_i_	The private key of U_i_
*P*_i_	The public key of U_i_
*ID*_i_	The identity of U_i_
*signature*_i_	The signature of U_i_
*MIC*_i_	The massage integrity code of the message generated by U_i_
⊕	The simple exclusive-OR operation
||	The message concatenation operation

## 3. The Proposed User Authentication Scheme

In this section, a user authentication scheme based on SCPK and ECC for a WANET is presented.

The proposed scheme is divided into three phases, namely the setup phase, the user registration phase, and the user authentication phase. In the setup phase, CA generates the system parameters and publishes them to users. In the user registration phase, users obtain their private/public key pairs by registering with CA. In the user authentication phase, users complete their identities authentication with the help of their private/public keys and the public key of CA.

The detail of the proposed user authentication scheme is described as follows.

### 3.1. The Setup Phase

We adopt an elliptic curve defined over GF(*p*) is recommended by SEC 2 [14]. First, the elliptic curve *E_p_*(*a*, *b*) over GF(*p*) is defined by y2=x3+ax+b (mod p), where *a* and *b* are real numbers, and $(4a^{3}+27b^{2})\mathrm{mod}p\neq 0.$ Next, a base point *G* = (*x_G_*, *y_G_*) with a very large value order is selected on *E_p_*(*a*, *b*). The order of *G*, *n*, is the smallest positive integer such that n⋅G=O, where *O* is infinity point. The global parameters of the system, (*p*, *a*, *b*, *G*, *n*), are known by all users in networks.

CA randomly chooses an integer $s_{\text{CA}}$, from [2, *n*−2] as its private key. In addition, CA’s paired public key is generated with:

(1) $${R^{\prime }}_{i}={r^{\prime }}_{i}\cdot G$$

And then, CA publishes *P*_CA_ to the whole network, but keeps $s_{\text{CA}}$ as a secret.

### 3.2. The User Registration Phase

When a user, U*_i_* with identity *ID_i_*, wants to join the system, he/she performs the following operations to register with CA.

First, U*_i_* generates a nonce, *N_i_*, using a pseudo-random number generator (PRNG), and randomly chooses an integer, $r_{i}^{\prime }$, from [2, *n*−2]. Then, U*_i_* computes:

(2) $$R_{i}^{\prime }=r_{i}^{\prime }\cdot G$$

And:

(3) $$ID_{i}^{\prime }=ID_{i}⊕SHA(r_{i}^{\prime }\cdot P_{\text{CA}})$$

After that, U*_i_* transmits *Message 1* $\left(N_{i},R_{i}^{\prime },ID_{i}^{\prime }\right)$ to CA. That is, ${\text{U}}_{i}\to \text{CA}:N_{i}||R_{i}^{\prime }||ID_{i}^{\prime }$.

Receiving $\left(N_{i},R_{i}^{\prime },ID_{i}^{\prime }\right)$ from U*_i_*, CA checks whether the message is fresh according to *N_i_*. If the message has been received, CA discards it and cancels the user registration. Otherwise, CA computes:

(4) $$SHA(s_{\text{CA}}\cdot R_{i}^{\prime })=SHA(s_{\text{CA}}\cdot r_{i}^{\prime }\cdot G)=SHA(r_{i}^{\prime }\cdot P_{\text{CA}})$$

The user’s identity is extracted by:

(5) $$ID_{i}=ID_{i}^{\prime }⊕SHA(s_{\text{CA}}\cdot R_{i}^{\prime })$$

CA checks *ID_i_*. If *ID_i_* has existed, CA cancels the user registration. Otherwise, CA randomly chooses an integer ${\tilde{r}}_{\text{CA}}$ from [2, *n*−2], and computes:

(6) $$R_{i}=R_{i}^{\prime }+{\tilde{r}}_{\mathrm{CA}}\cdot G$$

And:

(7) $${\tilde{s}}_{i}=(s_{\mathrm{CA}}\cdot SHA(ID_{i}||R_{i}.x)+{\tilde{r}}_{\mathrm{CA}})\mathrm{mod}n$$

where $R_{i}.x$ is the *x*-coordinate of the point *R_i_*.

CA generates a nonce, $N_{\text{CA}}$, using a PRNG, and returns *Message 2* $\left(N_{i},N_{\text{CA}},R_{i},{\tilde{s}}_{i}\right)$ to U*_i_*. That is, $\text{CA}\to {\text{U}}_{i}:N_{i}||N_{\text{CA}}||R_{i}||{\tilde{s}}_{i}$.

After receiving $\left(N_{i},N_{\text{CA}},R_{i},{\tilde{s}}_{i}\right)$ from CA, U*_i_* derives the private key as:

(8) $$s_{i}={\tilde{s}}_{i}+r_{i}^{\prime }=(s_{\text{CA}}\cdot SHA(ID_{i}||R_{i}.x)+{\tilde{r}}_{\text{CA}})\mathrm{mod}n+r_{i}^{\prime }$$

And U*_i_* verifies the authenticity of *P_i_* by:

(9) $$P_{i}=s_{i}\cdot G=P_{\mathrm{CA}}\cdot [(SHA(ID_{i}||R_{i}.x))\mathrm{mod}n]+R_{i}.$$

If this verification succeeds, U*_i_* accepts *P_i_* as his/her public key.

In the following, we demonstrate why the verification procedure described in (9) works correctly. According to Equations (6)–(8), we obtain:

$$\begin{array}{cc} s_{i}\cdot G & =[(s_{\text{CA}}\cdot SHA(ID_{i}||R_{i}.x)+{\tilde{r}}_{\text{CA}})\mathrm{mod}n+r_{i}^{\prime }]\cdot G\\ & =P_{\text{CA}}\cdot [SHA(ID_{i}||R_{i}.x)\mathrm{mod}n]+{\tilde{r}}_{\text{CA}}\cdot G+r_{i}^{\prime }\cdot G\\ & =P_{\text{CA}}\cdot [SHA(ID_{i}||R_{i}.x)\mathrm{mod}n]+R_{i}. \end{array}$$

Hence, U*_i_* computes $SHA(N_{\text{CA}}||r_{i}^{\prime }\cdot P_{\text{CA}})$ and returns *Message 3* ($SHA(N_{\text{CA}}||r_{i}^{\prime }\cdot P_{\text{CA}})$) to CA. That is, ${\text{U}}_{i}\to \text{CA}:SHA(N_{\text{CA}}||r_{i}^{\prime }\cdot P_{\text{CA}})$.

Receiving ($SHA(N_{\text{CA}}||r_{i}^{\prime }\cdot P_{\text{CA}})$), CA computes $SHA(N_{\text{CA}}||s_{\text{CA}}\cdot R_{i}^{\prime })$ and compares it with $SHA(N_{\text{CA}}||r_{i}^{\prime }\cdot P_{\text{CA}})$ received from U*_i_*. If $SHA(N_{\text{CA}}||s_{\text{CA}}\cdot R_{i}^{\prime })$ = $SHA(N_{\text{CA}}||r_{i}^{\prime }\cdot P_{\text{CA}})$, CA is be convinced that U*_i_* has verified the authenticity of his/her public key. Then, CA stores the registration information in the registration file. If $SHA(N_{\text{CA}}||s_{\text{CA}}\cdot R_{i}^{\prime })$ ≠ $SHA(N_{\text{CA}}||r_{i}^{\prime }\cdot P_{\text{CA}})$, CA cancels the user registration.

**Figure 2:**
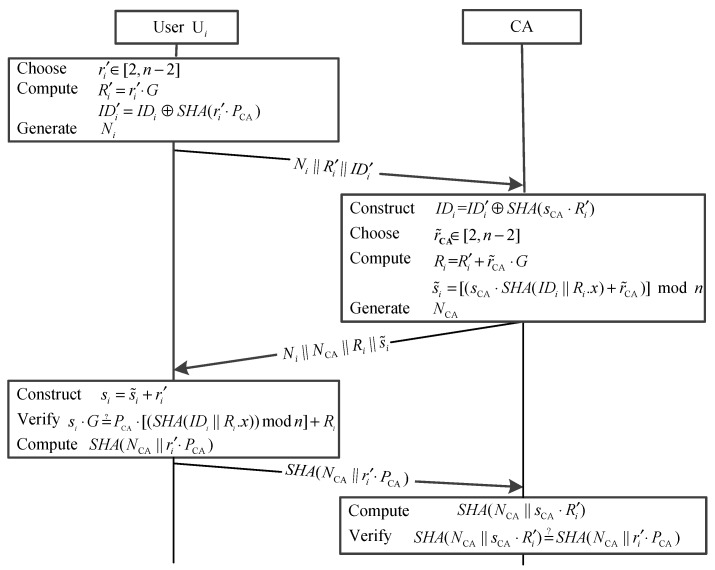
The user registration phase.

The interaction diagram of the user registration phase mentioned above is shown in Figure 2.

After U*_i_* finishes the registration successfully, he/she stores (*R_i_*, *ID_i_*, *s_i_*, *P_i_*). Other users can use *G*, *n*, *P*_CA_, *R_i_* and *ID_i_* to construct the public key of U*_i_*, *P_i_*.

### 3.3. The User Authentication Phase

The user authentication and session key agreement between Alice and Bob operates as follows, where Alice is an initiator and Bob is a responder.

Alice wants to set up a session key with Bob securely.

**Step 1:** Alice→Bob:NA||C|A|IDA||IDB||RA||signatureA

First, Alice generates a nonce, $N_{\text{A}}$, using a PRNG, and randomly chooses an integer, $r_{\text{A}}$, from [2,n−2]. Next, Alice computes $C_{\text{A}}=r_{\text{A}}\cdot G$ Then,Alice generates a signature using her private key as:

(10) $$signature_{\text{A}}=(r_{\text{A}}+s_{\text{A}}\cdot SHA(N_{\text{A}}||C_{\text{A}}||ID_{\text{A}}||ID_{\text{B}}||R_{\text{A}}))\mathrm{mod}n$$

Thereafter, Alice sends $\left(N_{\text{A}},C_{\text{A}},ID_{\text{A}},ID_{\text{B}},R_{\text{A}},signature_{\text{A}}\right)$ to Bob.

**Step 2:** $\text{Bob}\to \text{Alice}:N_{\text{A}}||N_{\text{B}}||C_{\text{B}}||ID_{\text{B}}||ID_{\text{A}}||R_{\text{B}}||MIC_{\text{B}}$

Receiving the message from Alice, Bob performs the following operations.

(1) According to $N_{\text{A}}$, Bob checks whether the message is fresh or not. If the message is fresh, Bob goes on the user authentication process. Otherwise, Bob rejects Alice’s authentication request.

(2) Bob computes Alice’s public key as:

(11) $$P_{\text{A}}=P_{\text{CA}}\cdot [(SHA(ID_{\text{A}}||R_{\text{A}}.x))\mathrm{mod}n]+R_{\text{A}}$$

Bob verifies the Alice’s signature as:

$$\begin{array}{cc} signature_{\text{A}}\cdot G & =[(r_{\text{A}}+s_{\text{A}}\cdot SHA(N_{\text{A}}||C_{\text{A}}||ID_{\text{A}}||ID_{\text{B}}||R_{\text{A}}))\mathrm{mod}n]\cdot G\\ & =r_{\text{A}}\cdot G+[(s_{\text{A}}\cdot SHA(N_{\text{A}}||C_{\text{A}}||ID_{\text{A}}||ID_{\text{B}}||R_{\text{A}}))\mathrm{mod}n]\cdot G\\ & =C_{\text{A}}+P_{\text{A}}\cdot [(SHA(N_{\text{A}}||C_{\text{A}}||ID_{\text{A}}||ID_{\text{B}}||R_{\text{A}}))\mathrm{mod}n]. \end{array}$$

If the signature is valid, Alice is a valid user and Bob continues the user authentication process. Otherwise, Bob cancels the user authentication process.

(3) Bob generates a nonce $N_{\text{B}}$, using a PRNG, and randomly chooses an integer $r_{\text{B}}$, from [2,n−2]. Next, Bob computes $C_{\text{B}}=r_{\text{B}}\cdot G$. Then, Bob computes the session key,

(12) $$K_{\text{BA}}=SHA((r_{\text{B}}+s_{\text{B}})\cdot (C_{\text{A}}+P_{\text{A}}))$$

and the message integrity code,

(13) $$MIC_{\text{B}}=SHA(K_{\text{BA}}||N_{\text{A}}||N_{\text{B}}||C_{\text{B}}||ID_{\text{B}}||ID_{\text{A}}||R_{\text{B}})$$

Finally, Bob sends $\left(N_{\text{A}},N_{\text{B}},C_{\text{B}},ID_{\text{B}},ID_{\text{A}},R_{\text{B}},MIC_{\text{B}}\right)$ to Alice.

**Step 3:** $\text{Alice}\to \text{Bob}:N_{\text{B}}||ID_{\text{A}}||ID_{\text{B}}||MIC_{\text{A}}$

Receiving the response from Bob, Alice executes the following operations.

(1) According to $N_{\text{A}}$, Alice checks whether the message is fresh or not. If the message is fresh, Alice continues the user authentication process. Otherwise, Alice cancels the user authentication process.

(2) Alice construct Bob’s public key as:

(14) $$P_{\text{B}}=P_{\text{CA}}\cdot [(SHA(ID_{\text{B}}||R_{\text{B}}.x))\mathrm{mod}n]+R_{\text{B}}$$

(3) Alice computes the session key as:

(15) $$K_{\text{AB}}=SHA((r_{\text{A}}+s_{\text{A}})\cdot (C_{\text{B}}+P_{\text{B}}))$$

and the message integrity code as:

(16) $$MIC_{\text{B}}^{\prime }=SHA(K_{\text{AB}}||N_{\text{A}}||N_{\text{B}}||C_{\text{B}}||ID_{\text{B}}||ID_{\text{A}}||R_{\text{B}})$$

Alice compares $MIC_{\text{B}}^{\prime }$ with $MIC_{\text{B}}$. If $MIC_{\text{B}}^{\prime }=MIC_{\text{B}}$, Alice passes the identity verification and regards Bob as a valid user.

Bob’s identity verification works as follows.

$$\begin{array}{cc} K_{\text{AB}} & =SHA((r_{\text{A}}+s_{\text{A}})\cdot (C_{\text{B}}+P_{\text{B}}))\\ & =SHA(r_{\text{A}}\cdot C_{\text{B}}+r_{\text{A}}\cdot P_{\text{B}}+s_{\text{A}}\cdot C_{\text{B}}+s_{\text{A}}\cdot P_{\text{B}})\\ & =SHA(r_{\text{A}}\cdot r_{\text{B}}\cdot G+r_{\text{A}}\cdot P_{\text{B}}+r_{\text{B}}\cdot P_{\text{A}}+s_{\text{A}}\cdot s_{\text{B}}\cdot G) \end{array}$$

$$\begin{array}{cc} K_{\text{BA}} & =SHA((r_{\text{B}}+s_{\text{B}})\cdot (C_{\text{A}}+P_{\text{A}}))\\ & =SHA(r_{\text{B}}\cdot C_{\text{A}}+r_{\text{B}}\cdot P_{\text{A}}+s_{\text{B}}\cdot C_{\text{A}}+s_{\text{B}}\cdot P_{\text{A}})\\ & =SHA(r_{\text{A}}\cdot r_{\text{B}}\cdot G+r_{\text{A}}\cdot P_{\text{B}}+r_{\text{B}}\cdot P_{\text{A}}+s_{\text{A}}\cdot s_{\text{B}}\cdot G) \end{array}$$

Hence, we have $K_{\mathrm{AB}}=K_{\text{BA}}$, and $SHA(K_{\text{AB}}||N_{\text{A}}||N_{\text{B}}||C_{\text{B}}||ID_{\text{B}}||ID_{\text{A}}||R_{\text{B}})=SHA(K_{\text{BA}}||N_{\text{A}}||N_{\text{B}}||C_{\text{B}}||ID_{\text{B}}||ID_{\text{A}}||R_{\text{B}})$ which implies the identity verification is valid.

(4) Alice computes $MIC_{\text{A}}=SHA(K_{\text{ AB}}||N_{\text{B}}||ID_{\text{A}}||ID_{\text{B}})$, and returns($N_{\text{B}},ID_{\text{A}},ID_{\text{B}}$, $MIC_{\text{A}}$) to Bob.

Receiving the message from Alice, Bob executes the following operations.

(1) According to $N_{\text{B}}$, Bob checks whether the message is fresh or not. If the message is fresh, Bob continues the user authentication process. Otherwise, Bob cancels the user authentication process.

(2) Bob computes $MIC_{\text{A}}^{\prime }=SHA(K_{\text{BA}}||N_{\text{B}}||ID_{\text{A}}||ID_{\text{B}})$, and compares it with $MIC_{\text{A}}=SHA(K_{\text{AB}}||N_{\text{B}}||ID_{\text{A}}||ID_{\text{B}})$ received from Alice. If $MIC_{\text{A}}^{\prime }=MIC_{\text{A}}$, Bob regards that Alice has verified his identity. At the same time, the session key agreement is successful, and the session key can be used for future communication.

Since $K_{\mathrm{AB}}=K_{\text{BA}}$, it is obvious that $MIC_{\text{A}}^{\prime }=MIC_{\text{A}}$.

**Figure 3:**
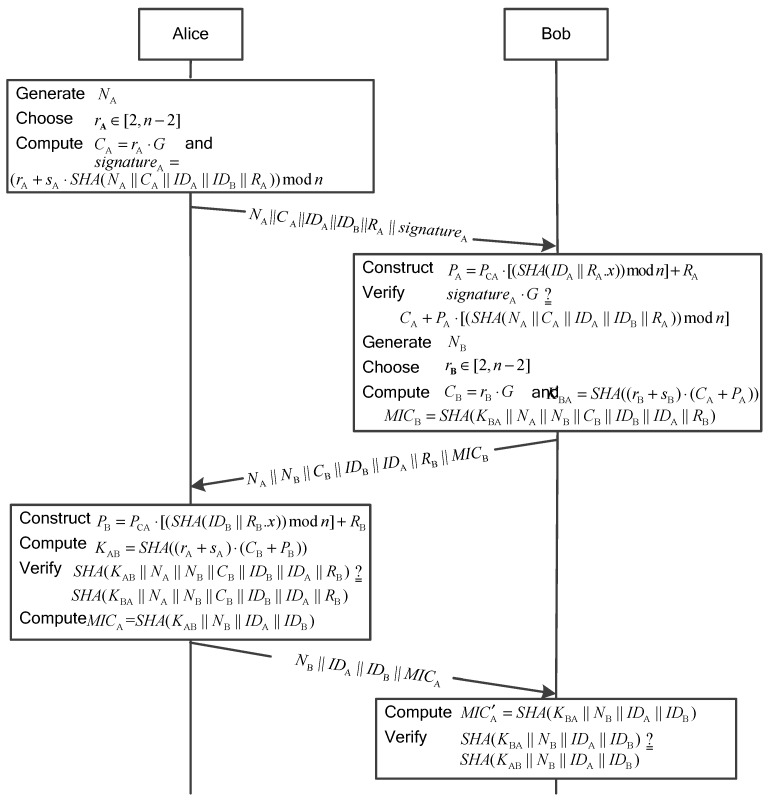
The user authentication phase.

The interaction diagram of the user authentication phase mentioned above is illustrated in Figure 3.

**Figure 4:**
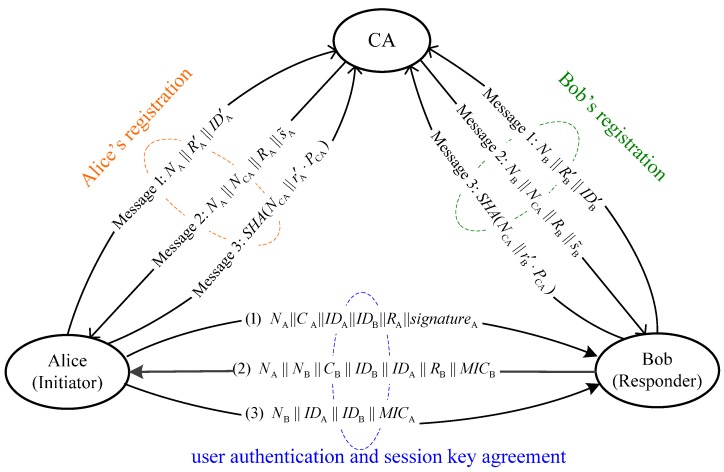
The proposed user authentication scheme.

The overall process of the proposed user authentication scheme is illustrated in Figure 4.

## 4. Security Analysis

The security of the proposed user authentication scheme is based on the intractability of reversing ECDLP and one-way hash function problem (OWHFP).

Let *E_p_*(*a*, *b*) be an elliptic curve over GF(p). *P* is a point with order *n* on the elliptic curve *E_p_*(*a*, *b*). *Q* is another point on the same curve.

The ECDLP is to determine *m* satisfying Q=m⋅P with given *P* and *Q*, which is difficult.

Let *h* be a one-way hash function. Given h(x), it is computationally infeasible to find *x.* Furthermore, for a given value *x* and h(x), it is computationally infeasible to find a *y* such that h(y)=h(x).

### 4.1. Security Analysis in User Registration Phase

**Theorem 1**. *The proposed user authentication scheme is secure against user masquerade attack, message-forgery attack, impersonate attack from CA in user registration phase*.

**Proof**.

(1) User masquerade attack resistance

We assume that an adversary (Eve) intercepts the legal user’s registration information and attempts to masquerade the legal user (${\text{U}}_{i}$) to join in the network. However, Eve will be faced with some difficulties in following scenarios.

Although Eve intercepts $ID_{i}^{\prime }$, he cannot masquerade the valid user. Because ${\text{U}}_{i}$ ’s identity is hidden in $ID_{i}^{\prime }=ID_{i}⊕SHA(r_{i}^{\prime }||P_{\text{CA}})$. If Eve wants to obtain $ID_{i}$ from $ID_{i}^{\prime }$, he must first obtain $SHA(r_{i}^{\prime }||P_{\text{CA}})$ which is protected under the OWHFP and ECDLP.

Although Eve intercepts message $\left(N_{i},N_{\text{CA}},R_{i},{\tilde{s}}_{i}\right)$ and wants to masquerade the valid user, he should derive $r_{i}^{\prime }$ from $R_{i}^{\prime }=r_{i}^{\prime }\cdot G$. It is not possible because solving the ECDLP is computationally infeasible. Meantime, he cannot return *Message 3* ($SHA(N_{\text{CA}}||r_{i}^{\prime }\cdot P_{\text{CA}})$) to CA without the knowledge of $r_{i}^{\prime }$.

Although Eve gets $ID_{i}$, he attempts to re-register with CA on the purpose of masquerading a valid user. Even if this attack is successful, the attack can be easily detected. This is because CA is convinced that the user has verified the authenticity of his public key since receiving Message 3. And CA stores the user’s registration information in the registration file. As a registration request is accepted, CA will check the submitted user’s identity information of the user in the registration file to prevent the re-registration attempt.

Therefore, our proposed scheme can resist the user masquerade attack.

(2) Message-forgery attack resistance

We assume that Eve intercepts $\left(N_{i},N_{\text{CA}},R_{i},{\tilde{s}}_{i}\right)$ when CA returns it to ${\text{U}}_{i}$ and attempts to forge $\left(R_{i},{\tilde{s}}_{i}\right)$.

${\text{U}}_{i}$ verifies the condition $s_{i}\cdot G=P_{\mathrm{CA}}\cdot [(SHA(ID_{i},R_{i}.x))\mathrm{mod}n]+R_{i}$. The verification does not hold because Eve needs to have the private key of CA, *P*_CA_. Hence, Eve should compute $s_{\text{CA}}$ from $P_{\text{CA}}=s_{\text{CA}}\cdot G$. It is not possible because solving the ECDLP is computationally infeasible. Therefore, our proposed scheme can resist the message-forgery attack.

(3) Resistance of the impersonate attack from CA

We assume that CA generates another pair of valid private/public key, ($s_{i}^{\prime },P_{i}^{\prime }$), satisfying (9), CA can impersonate ${\text{U}}_{i}$. However, this fraud can be detected by ${\text{U}}_{i}$ because two different valid keys exist. It can prove that CA is cheating. Therefore, our proposed scheme can resist the impersonate attack from CA.

### 4.2. Security Analysis in User Authentication Phase

**Theorem 2**. *The proposed user authentication scheme achieves mutual trust, and is secure against man-in-the-middle attack, replay attack, masquerading and tampering attacks in user authentication phase*.

**Proof**.

(1) Mutual trust

The signature of the message sent by Alice is generated in Step 1, which is verified by Bob in Step 2. In this way, Bob authenticates Alice’s identity.

Moreover, a message integrity code of the message sent by Bob, $MIC_{\text{B}}=SHA(K_{\text{BA}}||N_{\text{A}}||N_{\text{B}}||C_{\text{B}}||ID_{\text{B}}||ID_{\text{A}}||R_{\text{B}})$, is applied in Step 2. This provides the evidence of authentication and integrity for the message received by Alice. In the proposed scheme, $MIC_{\text{B}}$ contains $K_{\text{BA}}=SHA((r_{2}+s_{\text{B}})\cdot (C_{\text{A}}+P_{\text{A}}))$ generated by Bob’s private key. Hence, $MIC_{\text{B}}$ can be used to authenticate Bob’s identity.

Therefore, the proposed scheme provides the two-way authentication between Alice and Bob.

(2) Man-in-the-middle attack resistance

In the user registration phase, it prevents from the re-registration attempt so that adversaries can hardly masquerade other valid users to perform the man-in-the-middle attack.

In the user authentication phase, the proposed scheme exchanges $C_{\text{A}}=r_{\text{A}}\cdot G$ and $C_{\text{B}}=r_{\text{B}}\cdot G$ along with $signature_{\text{A}}$ and $MIC_{\text{B}}$, and generates the session keys, $K_{\text{AB}}=SHA((r_{\text{A}}+s_{\text{A}})\cdot (C_{\text{B}}+P_{\text{B}}))$ and $K_{\text{BA}}=SHA((r_{\text{B}}+s_{\text{B}})\cdot (C_{\text{A}}+P_{\text{A}}))$, using the private keys, $s_{\text{A}}$ and $s_{\text{B}}$, and two random values, $r_{\text{A}}$ and $r_{\text{B}}$. Man-in-the-middle attack is only possible if an adversary (Eve) can forge $signature_{\text{A}}$ and $MIC_{\text{B}}$. Hence, Eve must compute $s_{\text{A}}$ and $s_{\text{B}}$ from the pair ($P_{\text{A}}$, $P_{\text{B}}$) = ($s_{\text{A}}\cdot G$, $s_{\text{B}}\cdot G$). It is not possible because solving the ECDLP is computationally infeasible.

Therefore, the proposed scheme can resist man-in-the-middle attack.

(3) Replay attack resistance

Two types of replay attacks are considered. Type-I replay attack is defined as an adversary intercepts an authentication message and attempts to masquerade as a sender by replaying it without modifying any content of the authentication message. Type-II replay attack is defined as an adversary intercepts an authentication message and replays a forged authentication message modified from the original one.

Since the proposed scheme uses the nonce to ensure the fresh of message, the type-I replay attack will be excluded by checking the nonce. If Eve intercepts the message $\left(N_{\text{A}},C_{\text{A}},ID_{\text{A}},ID_{\text{B}},R_{\text{A}},signature_{\text{A}}\right)$ and replays it to impersonate Alice, Bob checks whether the message is fresh or not according to *N*_A_. If the nonce has been received, Bob discards the message.

In order to pass the authentication of Alice, Eve must change the nonce. It is assumed that Eve only changes the nonce from *N*_A_ to $N_{A}^{\prime }$ in $\left(N_{\text{A}},C_{\text{A}},ID_{\text{A}},ID_{\text{B}},R_{\text{A}},signature_{\text{A}}\right)$ to forge the authentication message. Bob verifies $signature_{\text{A}}\cdot G\;\overset{?}{=}C_{\text{A}}+P_{\text{A}}\cdot [(SHA(N_{A}^{\prime }||C_{\text{A}}||ID_{\text{A}}||ID_{\text{B}}||R_{\text{A}}))\mathrm{mod}n]$. The message verification does not hold since Eve needs to have the private key of Alice, *P*_A_, to generate a new signature. It is not possible because solving the ECDLP is intractable. In the same way, an adversary impersonating Bob cannot pass the authentication. Hence, the nonce cannot be forged in the proposed scheme, which means that the proposed scheme is also resistant to the type-II replay attack.

Therefore, the proposed scheme can resist the replay attack.

(4) Masquerading and tampering attacks resistance

It is assumed that an adversary (Eve) intercepts an authentication message and replays it to masquerade as a valid user.

Eve intercepts an authentication message sent by Alice and attempts to masquerade as Alice by launching the type-I replay attack. After Bob receives the authentication message, he will check whether the message is fresh or not according to *N*_A_. If the nonce has been received, Bob discards the message. On the other hand, Eve intercepts an authentication message and launches the type-II replay attack. It is difficult to succeed since Eve needs to use *P*_A_ to generate a new signature. Computing $s_{\text{A}}$ from $P_{\text{A}}\text{=}s_{\text{A}}\cdot G$ is not possible because solving the ECDLP is computationally infeasible.

It is assumed that an adversary (Eve) intercepts the message $\left(N_{\text{A}},C_{\text{A}},ID_{\text{A}},ID_{\text{B}},R_{\text{A}},signature_{\text{A}}\right)$ and attempts to tamper the message. This action will not pass the user authentication of Alice. As explained in the replay attack resistance, Eve needs to use *P*_A_ to generate a new signature. Hence, Eve encounters the intractability of solving the ECDLP. In addition, the one-way hash function is adopted in the user authentication phase to guarantee the integrity of message, which contains the session key generated by Alice and Bob’s private keys. Computing ($s_{\text{A}}$, $s_{\text{B}}$) from ($P_{\text{A}}$, $P_{\text{B}}$) is not possible because solving the ECDLP is computationally infeasible.

Therefore, the proposed scheme can resist the masquerading and tampering attacks.

**Theorem 3**. *Based on the difficulty in solving the ECDLP, the proposed user authentication scheme provides perfect forward secrecy, backward secrecy, key compromise impersonation attack resistance, known-key security, unknown key-share resistance, and known session-specific temporary information attack resistance*.

**Proof**.

(1) Perfect forward secrecy and backward secrecy

It is assumed that the private keys, $s_{\text{A}}$ and $s_{\text{B}}$, are compromised, and an adversary (Eve) attempts to compute the key $K_{\text{AB}}=SHA(r_{\text{A}}\cdot r_{\text{B}}\cdot G+s_{\text{B}}\cdot C_{\text{A}}+s_{\text{A}}\cdot C_{\text{B}}+s_{\text{A}}\cdot s_{\text{B}}\cdot G)$. Here, the forward secrecy is achieved by means of the term $r_{\text{A}}\cdot r_{\text{B}}\cdot G$. However, in order to compute the session key, Eve needs the knowledge of the random values, $r_{\text{A}}$ and $r_{\text{B}}$. Solving $C_{\text{A}}$ and $C_{\text{B}}$ to get $r_{\text{A}}$ and $r_{\text{B}}$ is equivalent to the problem of solving ECDLP.

In addition, the session key relies on the random values, $r_{\text{A}}$ and $r_{\text{B}}$, which are generated in each session independently and changed for each authentication phase.

Furthermore, another important aspect of our proposed scheme is that the session key is protected by the secure hash function. Although an adversary obtains a certain period session key, he/she cannot use the current session key to get forward and backward session keys. Hence, the session key in the proposed scheme achieves perfect forward secrecy and backward secrecy.

(2) Key compromise impersonation attack resistance

As defined in [15], the key compromise impersonation attack resistance is that an adversary (Eve) can masquerade as Alice if Alice’s private key is compromised, while Eve cannot masquerade as another user to interact with Alice.

It is assumed that the long-term private key of Alice, $s_{\text{A}}$, is compromised and known to Eve. Obviously, Eve can impersonate Alice using $s_{\text{A}}$. However, to impersonate any other user (Bob) to interact with Alice, Eve would need the session key, $K_{\text{BA}}=SHA(r_{\text{B}}\cdot C_{\text{A}}+r_{\text{A}}\cdot P_{\text{B}}+r_{\text{B}}\cdot P_{\text{A}}+s_{\text{A}}\cdot P_{\text{B}})$. Thus, Eve needs to have the private key of Bob, $s_{\text{B}}$, or the random value generated by Alice, $r_{\text{A}}$. Solving *P*_B_ and *C*_A_ to get $s_{\text{B}}$ and $r_{\text{A}}$ is equivalent to the problem of solving ECDLP. In addition, in most circumstances, the private key of a user is updated periodically.

Hence, the key compromise impersonation vulnerability can be limited to some considerably low extent.

(3) Known-key security

The proposed scheme achieves the known-key security if the knowledge of previous generated session keys does not allow an adversary to compromise the past or future session keys.

It is assumed that a session key generated by the proposed scheme is obtained by an adversary (Eve). Eve cannot derive all past and future session keys from the knowledge of the compromised session key. To derive a session key, Eve has to compute ($r_{\text{A}}$, $r_{\text{B}}$) and ($s_{\text{A}}$, $s_{\text{B}}$) from ($C_{\text{A}}$, $C_{\text{B}}$) and ($P_{\text{A}}$, $P_{\text{B}}$), respectively. It is not possible because solving the ECDLP is computationally infeasible.

(4) Unknown key-share resistance

A key agreement protocol achieves unknown key-share attack resistance if a user cannot be forced to share a session key with a different user rather than the one intended without their knowledge. That is, Alice cannot be forced to share a key with Eve when Alice believes that the key is shared with Bob.

In the user authentication phase of the proposed scheme, Bob sends a message to Alice, $N_{\text{A}}||N_{\text{B}}||C_{\text{B}}||ID_{\text{B}}||ID_{\text{A}}||R_{\text{B}}||MIC_{\text{B}}$. And *MIC*_B_ contains $K_{\text{BA}}=SHA((r_{\text{B}}+s_{\text{B}})\cdot (C_{\text{A}}+P_{\text{A}}))$ generated by Bob’s private key, $s_{\text{B}}$. Similarly, Alice responds to Bob with the message, $N_{\text{B}}||ID_{\text{A}}||ID_{\text{B}}||MIC_{\text{A}}$. And *MIC*_A_ contains $K_{\text{AB}}=SHA((r_{\text{A}}+s_{\text{A}})\cdot (C_{\text{B}}+P_{\text{B}}))$ generated by Alice’s private key $s_{\text{A}}$. The verification of *MIC*_B_ and *MIC*_A_ at Alice and Bob confirms the generation of same session key. 

Therefore, the proposed scheme resists the unknown key-share attack.

(5) Known session-specific temporary information attack resistance

The security of the generated session key should not be compromised even if two random values are compromised by an adversary (Eve).

In the proposed scheme, Eve cannot derive the session key $K_{\text{AB}}=SHA((r_{\text{A}}+s_{\text{A}})\cdot (C_{\text{B}}+P_{\text{B}}))$ and $K_{\text{BA}}=SHA((r_{\text{B}}+s_{\text{B}})\cdot (C_{\text{A}}+P_{\text{A}}))$ even if $r_{\text{A}}$ and $r_{\text{B}}$ are compromised. This is because Eve does not know Alice’s private key and Bob’s private key, $s_{\text{A}}$ and $s_{\text{B}}$. Moreover, Eve cannot derive from ($P_{\text{A}}$, $P_{\text{B}}$)=($s_{\text{A}}\cdot G$, $s_{\text{A}}\cdot G$) because solving the ECDLP is computationally infeasible.

Therefore, the proposed scheme resists the known session-specific temporary information attack. 

## 5. Performance Analysis

In this section, we analysis the performance of the proposed user authentication scheme, in terms of security, storage overhead, communication overhead and computation overhead.

(1) Attack resistance and functionality

The attack resistance and functionality of the proposed user authentication scheme are compared with other three schemes, namely Diffie-Hellman key agreement scheme in [4] (abbreviated as DHKA scheme), the user authentication phase of secure MAC protocol for cognitive radio networks in [5] (abbreviated as SecureMAC protocol), and authentication and key agreement scheme in [11] (abbreviated as AKA scheme).

The comparison results are listed in Table 2. From Table 2, we observe that our proposed user authentication scheme provides two-way user authentication and session key agreement. However, SecureMAC protocol in [5] does not achieve the session key agreement.

**Table 2:** The functionality comparison.

Functionality	DHKA Scheme in [4]	SecureMAC Protocol in [5]	AKA Scheme in [11]	Proposed Scheme
Mutual trust	Yes	Yes	Yes	**Yes**
Session key agreement	Yes	No	Yes	**Yes**
Time synchronization	Not need	Not need	Need	**Not need**
Replay attack resistance	No	Yes	Yes	**Yes**
Man-in-the middle attack resistance	Yes	Yes	Yes	**Yes**
Forward secrecy	No	No	Yes	**Yes**
Backward secrecy	No	No	Yes	**Yes**
Key compromise impersonation attack resistance	No	No	No	**Yes**

Moreover, the session key of our proposed scheme achieves perfect forward secrecy and backward secrecy, and key compromise impersonation attack resistance compared with DHKA scheme in [4] and AKA scheme in [11].

In addition, our proposed scheme also defends against the replay attack with modified challenge-response mechanism, but DHKA scheme in [4] is vulnerable to the replay attack. AKA scheme in [11] defends against the replay attack using timestamp mechanism.

(2) Storage overhead

Each user needs store parameters (*p*, *a*, *b*, *G*, *n*, *P*_CA_, *R_i_*, *ID_i_*) and the private/public key pair (*s_i_*, *P_i_*). In our proposed scheme, we assume that the key length of ECC is 160 bits, and the length of ID value is 160 bits. The storage overhead of each user is listed in Table 3.

**Table 3:** Storage overhead of each user.

Parameters	Storage Overhead (bits)
The parameters of ECC, (*p*, *a*, *b*, *G*, *n*)	960/(160 + 160 + 160 + 320 + 160)
CA’s public key, *P*_CA_	320
Point *R_i_*	320
User identity, *ID_i_*	160
User’s private key, *s_i_*	160
User’s public key, *P_i_*	320
**Total**	**2240**

The total storage overhead is only 2,240 bits, which is quite suitable for resource-constrained wireless network.

For security, the private key of U*_i_*, *s_i_*, needs to be stored in the form of ciphertext, and the public key of U*_i_*, *P_i_*, and other parameters, (*p*, *a*, *b*, *G*, *n*, *P*_CA_, *R_i_*, *ID_i_*) are stored in the form of plaintext. Since other users can use *n*, *P*_CA_, *R_i_* and *ID_i_* to construct the public key of U*_i_*, *P_i_*, users does not need to store the public keys of other users with whom he/she is communicating. In addition, since the generated session key between two users is temporary, it does not need to be stored.

(3) Communication overhead

Let the length of nonce be 64 bits, and the hash value of the one way hash function is 256 bits. The communication overhead in the user authentication phase of our proposed scheme is listed in Table 4.

**Table 4:** Communication overhead of each user.

Message	Communication Overhead (bits)
Step 1	1184
Step 2	1344
Step 3	640
**Total**	**3168**

From Table 4, it is obvious that the communication overhead in the user authentication phase of our proposed scheme is relatively light.

(4) Computation overhead

The computational complexity is analyzed in detail and compared with some other user authentication schemes, namely DHKA scheme in [4], AKA scheme in [11], time stamp mechanism and key management scheme in [16] (abbreviated as TSMKM scheme), authentication scheme based on bilinear pairings) in [17] (abbreviated as BP-A scheme), ECC-based authentication key agreement scheme in [18] (abbreviated as ECC-AKA scheme), and ECC-based improved authentication key agreement scheme in [19] (abbreviated as ECC-IAKA scheme).

The notations of various operations and the denotations used in this subsection are listed in Table 5.

**Table 5:** Definition of various operations.

Notations	Denotations
$T_{\text{EM}}$	The time for computing a point multiplication on GF(p)
$T_{\text{EA}}$	The time for computing a point addition on GF(p)
$T_{\text{BP}}$	The time for computing a bilinear pairing
$T_{\text{MI}}$	The time for computing modular inversion
$T_{\text{MM}}$	The time for computing modular multiplication
$T_{\text{MA}}$	The time for computing modular addition
$T_{\text{ME}}$	The time for computing modular exponentiation
$T_{\text{H}}$	The time for computing the one-way hash function
$T_{\text{RSA-Ver}}$	The time for computing RSA signature verification operation
$T_{\text{X}}$	The time for computing symmetric encryption/decryption operation

According to [19,20,21,22,23], $T_{\text{BP}}\approx 3T_{\text{EM}}$, $T_{\text{EM}}\approx 29T_{\text{MM}}$, $T_{\text{EA}}\approx 0.12T_{\text{MM}}$, $T_{\text{ME}}\approx 240T_{\text{MM}}$, and $T_{\text{MI}}\approx 3T_{\text{MM}}$. Compared to the computational time for performing other operations, the time for performing the modular addition and one-way hash function can be negligible. The comparison of computation overhead is listed in Table 6.

As shown in Table 6, our proposed user authentication scheme does not involve modular exponentiation and bilinear pairing operations, while DHKA scheme in [4] and the BP-A scheme in [17] require two modular exponentiation operations and three bilinear pairing operations, respectively. Meanwhile, our proposed scheme reduces the amount of point multiplication operations compared with the AKA scheme in [11], the TSMKM scheme in [16] and the ECC-IAKA scheme in [19]. The ECC-AKA scheme in [18] utilizes both RSA and ECC to achieve mutual authentication, which increases the computation burden on user’s side. Hence, the computation overhead of our proposed scheme is obviously less than that of other compared schemes.

**Table 6:** Computation overhead of each user.

Schemes	Computation Overhead	Equivalent Computation Overhead
DHKA scheme in [4]	$2T_{\text{ME}}$	$480T_{\text{MM}}$
AKA scheme in [11]	$15T_{\text{EM}}+4T_{\text{MM}}+4T_{\text{MA}}+6T_{\text{EA}}+T_{\text{MI}}+6T_{\text{H}}$	$442.72T_{\text{MM}}$
TSMKM scheme in [16]	$15T_{\text{EM}}+5T_{\text{EA}}+2T_{\text{MI}}+4T_{\text{MM}}+T_{\text{MA}}+8T_{\text{H}}$	$445.6T_{\text{MM}}$
BP-A scheme in [17]	$2T_{\text{EM}}+3T_{\mathrm{BP}}+8T_{\text{H}}$	$319T_{\text{MM}}$
ECC-AKA scheme in [18]	$10T_{\text{EM}}+4T_{\text{EA}}+8T_{\text{MA}}+8T_{\text{MM}}+4T_{\text{MI}}+10T_{\text{H}}+2T_{\text{RSA-Ver}}$	$310.48T_{\text{MM}}+2T_{\text{RSA-Ver}}$
ECC-IAKA scheme in [19]	$17T_{\text{EM}}+5T_{\text{EA}}+3T_{\text{H}}+T_{\text{X}}$	$493.6T_{\text{MM}}+T_{\text{X}}$
**Our proposed scheme**	$8T_{\text{EM}}+5T_{\text{EA}}+3T_{\text{MA}}+T_{\text{MM}}+10T_{\text{H}}$	$233.6T_{\text{MM}}$

Moreover, as the performance analysis in [24], some parameters can be pre-computed to reduce the computational complexity. In our proposed scheme, *C*_A_ and *C*_B_ can be computed in advance. In this way, the computational complexity can be reduced in some extent.

In addition, if some applications require lower computational complexity, a higher clock frequency for hardware implementations or binary-field based elliptic curves [25] can be selected for our proposed scheme.

## 6. Conclusions

The WANET will play an important role in the next generation wireless networking. In addition, security issue is critical to deploy and manage WANETs. Furthermore, the user authentication is the first safety barrier in a network.

We proposed a user authentication scheme based on SCPK and ECC for the WANET, in which an efficient two-way user authentication and a secure session key agreement are achieved. Based on the security and performance analysis, our proposed scheme resists various common known attacks, such as man-in-the-middle attack, replay attack, masquerading and tampering attacks, as well as achieves lower storage, communication, and computation overheads. Therefore, the proposed user authentication scheme based on SCPK and ECC is efficient and suitable for the resource-constrained WANET.

## References

[1] Kumaar S.S., Mangai M., Fernado N., Daniel J.V. (2013). A survey of various attacks in mobile ad hoc networks. Int. J. Comput. Sci. Mob. Comput..

[2] Bechler M., Hof H.J., Kraft D., Pahlke F., Wolf L. A cluster-based security architecture for ad hoc networks. Proceedings of the 23th Annual Joint Conference of the IEEE Computer and Communications Societies (INFOCOM 2004).

[3] Deng H., Mukherjee A., Agrawal D.P. Threshold and identity-based key management and authentication for wireless ad hoc networks. Proceedings of Information Technology: Coding and Computing (ITCC 2004).

[4] Zhu X., Xu S. A new authentication scheme for wireless ad hoc network. Proceedings of the 2012 International Conference on Information Management, Innovation Management and Industrial Engineering (ICIII 2012).

[5] Alhakami W., Mansour A., Safdar G.A., Albermany S. A secure MAC protocol for cognitive radio networks (SMCRN). Proceedings of the 2013 Science and Information Conference (SAI 2013).

[6] Kohnfelder L. (1978). Towards a Practical Public-Key Cryptosystem. Ph.D. Thesis.

[7] Shamir A. Identity-based cryptosystems and signature schemes. Proceedings of Advances in Cryptology (CRYPTO 84).

[8] Girault M. Self-certified public keys. Proceedings of Advances in Cryptology (EUROCRYPT 91).

[9] Johnson D., Menezes A., Vanstone S. (2001). The elliptic curve digital signature algorithm (ECDSA). Int. J. Inf. Secur..

[10] Jing C., Li B., Xu H. An efficient scheme for user authentication in wireless sensor networks. Proceedings of the 21th IEEE International Conference on Advanced Information Networking and Applications Workshops (AINAW 2007).

[11] Zhao X., Lv Y., Yeap T.H., Hou B. A novel authentication and key agreement scheme for wireless mesh networks. Proceedings of the 5th IEEE International Joint Conference on INC, IMS and IDC (NCM 2009).

[12] Zhang C., Wang X. A novel self-certified security access authentication protocol in the space network. Proceeding of the 2012 IEEE International Conference on Communication and Technology (ICCT 2012).

[13] Petersen H., Horster P. (1997). Self-certified keys concepts and applications. Commun. Multimed. Secur..

[14] Daniel R.L. (2012). Standards for Efficient Cryptography, SEC 2: Recommended Elliptic Curve Domain Parameters.

[15] Giruka V., Chakrabarti S., Singhal M. (2006). A distributed multi-party key agreement protocol for dynamic collaborative groups using ECC. J. Parallel Distrib. Comput..

[16] Indra G., Taneja R. (2014). A time stamp-based elliptic curve cryptosystem for wireless ad-hoc sensor networks. Int. J. Space-Based Situat. Comput..

[17] Zhang J., Li X., Ma J., Wang W. (2014). Secure and efficient authentication scheme for mobile sink in WSNs based on bilinear pairings. Int. J. Distrib. Sens. Netw..

[18] Ammayappan K., Negi A., Sastry V., Das A. (2011). An ECC-based two-party authenticated key agreement protocol for mobile ad hoc networks. J. Comput..

[19] Li X., Wen Q., Zhang H., Jin Z. (2013). An improved authentication with key agreement scheme on elliptic curve cryptosystem for global mobility networks. Int. J. Netw. Manag..

[20] Wu T., Hsu C., Lin H. (2009). Self-certified multi-proxy signature schemes with message recovery. J. Zhejiang Univ..

[21] Babamir F.S., Norouzi A. (2014). Achieving key privacy and invisibility for unattended wireless sensor networks in healthcare. Comput. J..

[22] Holbl M., Welzer T., Brumen B. (2012). An improved two-party identity-based authenticated key agreement protocol using pairings. J. Comput. Syst. Sci..

[23] Tsaur W.J., Yeh Y. (2011). A novel mobile agent authentication scheme for multi-host environments using self-certified pairing-based public key cryptosystem. Int. J. Innov. Comput. Inf. Control.

[24] Jiang Y., Lin C., Shen X., Shi M. (2006). Mutual authentication and key exchange protocols for roaming services in wireless mobile networks. IEEE Trans. Wirel. Commun..

[25] Wenger E. Hardware architectures for MSP430-based wireless sensor nodes performing elliptic curve cryptography. Proceedings of the 11th International Conference on Applied Cryptography and Network Security (ACNS 2013).

